# Exploring associations between sprinting mechanical capabilities, anaerobic capacity, and repeated-sprint ability of adolescent ice hockey players

**DOI:** 10.3389/fspor.2023.1258497

**Published:** 2023-12-12

**Authors:** Julien Glaude-Roy, Philippe Pharand, Jean-François Brunelle, Jean Lemoyne

**Affiliations:** ^1^ Department of Human Kinetics, Université du Québec à Trois-Rivières, Trois-Rivières, QC, Canada; ^2^ Laboratoire de Recherche sur le Hockey, Université du Québec à Trois-Rivières, Trois-Rivières, QC, Canada; ^3^ Service de l'Activité Physique et Sportive, Université du Québec à Trois-Rivières, Trois-Rivières, QC, Canada

**Keywords:** anaerobic capacity, repeated-sprint ability, speed, power, force-velocity profile, performance

## Abstract

**Introduction:**

Sprinting ability and anaerobic capacities are the determinant variables of the performance of ice hockey players. Recent research in sprinting showed the existence of distinct force–velocity (*F*–*V*) profiles, but the link between these profiles and anaerobic capacities remains unclear. This study explores the associations between *F*–*V* variables and anaerobic capacities among cohorts of highly trained adolescent ice hockey players.

**Methods:**

Data from 36 men (age, 15.1 ± 0.2 years) and 34 women (age, 16.5 ± 0.7 years) were collected during off-season camps. All athletes completed a 30-m sprint test, a Wingate anaerobic test (WAnT), and a repeated-sprint anaerobic (RSA) test. *F*–*V* variables were calculated from the 30-m sprint test. Partial Pearson correlation coefficients for pooled data and Pearson correlation coefficients for individual male and female datasets were calculated.

**Results:**

Among the *F*–*V* variables, maximal theoretical velocity and power were moderately to largely associated with WAnT and RSA performance (|*r*| = 0.30–0.70). Maximal theoretical force was moderately associated with the RSA (*r* = −0.32 to −0.33).

**Discussion:**

The results indicate the importance for highly trained adolescent players to be able to apply force at high velocities to maximize anaerobic capacities. Important differences between male and female players suggest training priorities may differ according to sex.

## Introduction

With approximately 18% of effective playing time spent in high-intensity skating activities, performance in ice hockey is a combination of short bouts of explosive and powerful movements cumulating in high energetic demands (1). The ability to skate and accelerate at higher speeds distinguishes the best young players (2). Improving sprinting ability off the ice during off-season is important as it may improve skating performance in-season (3). Developing the anaerobic system is also important as this metabolic system assumes 69% of energy costs during competition (4). To develop both of these capacities, strength and conditioning coaches turn to combinations of various off-ice methods, such as strength exercises, heavy resisted sprints, repeated sprints, and high-intensity training (5–9). However, the concurrent use of all these training methods during off-season presents periodization challenges that may hinder strength/power and anaerobic gains (10, 11).

In recent years, analysis of the sprint force–velocity (*F*–*V*) profile has gained popularity among researchers and practitioners who have turned toward this evaluation method to efficiently individualize training prescriptions (12, 13). The procedure gives an overall view of the strength/power/velocity capabilities of an athlete (defined in Table 1) during sprinting tasks by following the time–velocity curve of their center of mass (14). The mechanical capabilities evaluated using this method for a given athlete are compared with those of an optimal profile (12, 15). In fact, Morin and Samozino (15) have demonstrated that theoretical maximal force (*F*_0_) translates to shorter distance sprint performance (<20 m), whereas theoretical maximal velocity (*V*_0_) translates to longer distance sprint performance (>30 m).

**Table 1:** Definitions for *F*–*V*, WAnT, and RSA test variables.

Test	Variable	Definition	Units
*F*–*V*	*F*_0_	Maximal theoretical horizontal force relative to body mass	N kg^−1^
	*V*_0_	Maximal theoretical horizontal velocity	M s^−1^
	*P*_max_	Maximal theoretical horizontal power relative to body mass	W kg^−1^
	*S* **_fv_**	Slope of the force–velocity relationship applied to the center of mass	N m s^−1^
	**S_fvopt_**	Slope of the force–velocity relationship applied to the center of mass based on the maximal theoretical horizontal power and best performance of athletes at 10 m.	N m s^−1^
	*FV*_imb._	Represents the relative difference between *S*_fv_ and *S*_fvopt_ computed as $100\cdot \left(1-\frac{S_{\mathrm{fv}}}{S_{\mathrm{fvopt}}}\right)$. A positive value is interpreted as a force deficit and a negative value as a velocity deficit.	%
	*D_rf_*	Rate of decrease of the ratio of the horizontal component of the ground-reaction force to the corresponding resultant force	%
	*RF*_max_	Maximal ratio of the horizontal component of the ground-reaction force to the corresponding resultant force	%
Wingate	Peak power	Maximal average power recorded during one of the six 5-s windows	W
	Relative peak power	Maximal average power recorded during one of the six 5-s windows relative to body mass.	W kg^−1^
	Average power	Sum of the six 5-s average power windows divided by total power output	W
	Relative average power	Sum of the six 5-s average power windows divided by total power output relative to body mass.	W kg^−1^
	Anaerobic capacity	Sum of the six 5-s average power windows	W
	Anaerobic fatigue	Rate of decrease of power output.	%
RSA	Best time	Best time of first two repetitions in the RSA anaerobic sprint test	s
	Total time	Time sum off all six sprints performed in the RSA anaerobic sprint test	s
	Performance decrement score	Rate of performance decrease in sprint time.	%

Available research including ice hockey players focused mainly on associations between sprinting *F*–*V*, skating *F*–*V*, vertical jump performance, and skating performance. Perez et al. (16) explored the associations between both profiles and jumping, running, or skating performance with 17 French national team female players (mean age, 21.6 ± 3.4 years). They demonstrated that maximal theoretical power (*P*_max_) displayed the strongest associations with the three performance indicators (|*r*| ranging from 0.81 to 0.92; *p* < 0.001). Other work including ice hockey players was used to confirm the validity and reliability of the method on the ice, this time with French elite female players and Finnish elite male junior players (17–19). To our knowledge, *F*–*V* data for highly trained adolescent ice hockey players have yet to be published. As the physical and physiological performance does not linearly change during the critical stage of adolescence (20–22), studying these age groups is important. Understanding the links between sprinting *F*–*V* and a wider set of physical or physiological markers such as anaerobic capacity could guide the decisions of the practitioners during off-season.

In other sports, some work has linked sprint mechanical capabilities to anaerobic capacities. The results with rugby sevens players and soccer players consistently showed that the ability to efficiently generate force at high velocities (e.g., *V*_0_ and *D*_rf_) was impaired while the ability to efficiently generate force at low speeds (e.g., *F*_0_ and *Rf*_max_) remained constant during repeated sprints (23, 24). In both cases, linear sprints were used during the repeated-sprint test even though team sports performance includes much shorter sprints and multiple changes of directions (25). In addition to such limitations, the links between mechanical capabilities during an independent maximal sprint test and repeated-sprint ability were not reported. Given the widespread use of anaerobic capacity tests during off-season testing of ice hockey players (25, 26), a better understanding of the associations between sprinting *F*–*V* and common anaerobic capacity tests could help practitioners narrow down the preferred and most effective training methods during off-season. Hence, this study aimed to explore associations between sprinting *F*–*V* mechanical capabilities and anaerobic capacities of 15- to 17-year-old male and female ice hockey players.

Two specific objectives arise from this study. First, this study explores how the mechanical capabilities of sprinting *F*–*V* are related to the variables from anaerobic tests used in ice hockey (e.g., the Wingate and a repeated-sprint ability test with a 180° turn). Second, this study explores the differences between male and female players. Based on the previous results by Perez et al. (16), it was hypothesized that *P*_max_ shows the strongest associations with the different variables of both anaerobic tests. It was also believed that *V*_0_ shows stronger associations with the Wingate given the time needed to reach peak power (around 7 s) and *F*_0_ shows stronger associations with the repeated sprints due to the included change of direction in this study. Finally, given that female athletes tend to have more force-oriented profiles (27, 28), it was hypothesized that *F*_0_ plays a more important role in the anaerobic performance for this population.

## Methods

### Participants

Every spring, the Quebec Ice Hockey Federation invites the most promising players (men and women) to development camps. Invitations are based on the performance of players during the regular season. Among these invited players, the best will be part of the team competing at the national level. In the summer of 2022, 42 men and 42 women were invited to a summer camp launching off-season training for players. Various physical and physiological abilities were tested. Goaltenders were excluded from the study design given the unique nature and demands of their position (29). Injured players or players who were unable to complete the entire testing protocol were also excluded from the study. Finally, 36 men (age = 15.1 ± 0.2 years, stature = 177.5 ± 6.4, mass = 70.6 ± 8.4) and 34 women (age = 16.5 ± 0.7 years, stature = 167.3 ± 5.5, mass = 65.4 ± 7.0) participated in the study. Given their training status and preparation for national-level competitions, the participants are classified as highly trained athletes (30). *Post hoc* power analysis for between-groups comparisons and correlations was computed using G*Power v.3.1.9.7. Statistical power for the smallest observed effect size (0.67) at a significance level of *α* = 0.05 was 0.8 for both methods. This study was approved by the ethics board of the researchers’ institution (CER-21-278-07.29). Following the rules and regulations of our country and province, only written consent from players was obtained. Article 21 of the Civil Code of Quebec stipulates that “Consent to research that could interfere with the integrity of a minor may be given by the person having parental authority or the tutor. A minor 14 years of age or over, however, may give consent alone if, in the opinion of the competent research ethics committee, the research involves only minimal risk and the circumstances justify it.”

### Study design

Male and female players were tested on two separate consecutive days, following similar procedures (e.g., Day 1 = men, Day 2 = women). The players were divided into two subgroups alternating between physical testing and team meetings. The testing protocol of the camp consisted of anthropometric measurements, strength, speed, agility, power, anaerobic capacity, and mobility. These physical parameters were collected via various tests: stature, mass, grip strength, chin-ups, bench press, standing long jump, countermovement jump, 30-s Wingate anaerobic test (WAnT), 30-m sprint, 5-10-5 shuttle run, and repeated anaerobic sprint test with a 180° change of direction [repeated-sprint anaerobic (RSA)]. The tests were divided into two sessions. For the morning session, the players were divided into five groups alternating between one agility and five muscular tests. The WAnT was done at the end of the session. The players then had 3 h before the afternoon session. During this session, they completed the 30-m sprint followed 15 min later by the RSA.

### Horizontal *F*–*V* profile

To determine the individual *F*–*V* of each player, the players ran two 30-m sprints. The method has an “acceptable” intraday and inter-day reliability [intraclass correlation coefficient (ICC) ≥ 0.75 and coefficient of variation (CV) ≤ 10%] (14). Before the sprints, the players started in a feet supported crouched position and stood still until instructed to run by the research assistant. They were given 3- to 4-min rest between trials. Instant velocity over the sprinting distance was collected at 46.875 Hz using a Stalker ATS II (Stalker Sport, Richardson, TX, USA). The radar was placed 3 m behind the starting line and 1 m aboveground approximately at the height of the center of mass of the players. Following protocols and guidelines established by Samozino et al. (14), raw velocity data were first imported into *R* studio. Then, data prior to the start and after the maximal velocity plateau were discarded. The remaining data were fitted with a mono-exponential function, and mechanical variables were calculated (14, 18). An optimal profile at 10 m (*S*_fvopt_) was computed following the proposed methods by Samozino et al. (31). To improve reliability, the average values of both trials were used for further analyses (32, 33).

### Wingate

To evaluate anaerobic capacity, the WAnT was used. The WAnT is a popular test that measures the power production of a player throughout a 30-s bout. The validity and reliability of the test to evaluate the anaerobic capacity of ice hockey players were confirmed (34, 35). The test was completed on an air-braked cycle ergometer (Wattbike Pro/Trainer, Nottingham, UK, 2022), which was also validated in a previous study (33). The test started with a 5-min warm-up on a cycle ergometer with a resistance of 75 W and a cadence of 85 revolutions per minute (rpm). For the last 6 s of each minute, the players were asked to sprint. A 3-min rest period was given before the start of the test. After the WAnT, a 2-min cooldown was given. Peak power, relative peak power, average power (e.g., average power exerted during the test), relative average power, anaerobic capacity (e.g., the sum of average values recorded in each of six 5-s blocks), and anaerobic fatigue (e.g., drop-off in power from the highest to the lowest 5-s block represented as a percentage) were extracted directly from the Wattbike cycle ergometer console and used for analysis.

### Repeated-sprint ability

To evaluate the anaerobic capacity of the players in a more practical approach, a RSA test with a 180° change of direction (RSA) was designed. In practice, the available space was limited and imposed constraints for the chosen test format in line with guidelines by Kyles et al. (25). The players ran 20 m and then changed direction to run back 20 m. The players completed six sprints and had 30 s to complete a sprint and start the next. This procedure was validated previously with cohorts of soccer players (36) and chosen to introduce a change of direction component, which is important for the performance in team sports such as ice hockey. Sprint times were collected using Swift (Swift Performance, Northbrook, IL, USA) single-beam laser timing gates placed on the starting line. The players started 30 cm behind the gates. The best time between the first two sprints, total time, and a fatigue index were used for analysis. The fatigue index was computed following the recommendations of Glaister et al. (37) by using the percentage decrement score [100 × (total sprint time ÷ ideal sprint time)] − 100, where the ideal sprint time = number of sprints × fastest sprint time. The results for two female players and seven male players were excluded from final analysis as they did not perform their best sprint in the first two trials, which could indicate the use of a pacing strategy (24).

### Statistical analysis

Descriptive statistics for sprinting *F*–*V*, Wingate, and RSA are presented in Table 1. Normality assumptions for each variable were confirmed by verifying the skewness and kurtosis of each distribution. To compare male and female players, independent Student *t*-test were computed with Cohen's D to estimate effect sizes. The reliability of *F*–*V* mechanical capabilities with adolescent ice hockey players was confirmed by computing ICCs and CV for both trials. All variables had ICC values ≥ 0.90 and CV ≤ 10% (38). To explore relations between the *F*–*V* and anaerobic capacity of the players while controlling for sex differences, partial Pearson correlation coefficients for pooled data and Pearson correlation coefficients for individual sex (e.g., men and women) were calculated. As recommended by Hopkins et al. (38), the magnitude of Pearson's correlation coefficients (*r*) was considered trivial (*r* < 0.10), small (*r* = 0.10–0.29), moderate (*r* = 0.30–0.49), large (*r* = 0.50–0.69), very large (*r* = 0.70–0.89), nearly perfect (*r* = 0.90–0.99), and perfect (*r* = 1.00). Statistical analyses were conducted using SPSS version 28.0 (IBM Corporation, Armonk, NY, USA).

## Results

### Associations between *F*–*V* mechanical capabilities and anaerobic capacities

Partial Pearson's correlation coefficients suggest that *F*_0_ had a small association with the relative peak power (*r* = 0.26, *p* = 0.049) of the WAnT and was moderately associated with total time (*r* = −0.32, *p* = 0.012) and best time (*r* = −0.33, *p* = 0.10) of the RSA. *V*_0_ and *P*_max_ were moderately associated with peak power (*r* = 0.43 and 0.35, *p* < 0.001 and *p* = 0.006), relative peak power (*r* = 0.49 and 0.44, *p* < 0.001), average power (*r* = 0.30, *p* = 0.019 and 0.018), and anaerobic capacity (*r* = 0.37 and 0.32, *p* = 0.003 and 0.012) of the WAnT and were largely associated with total time (*r* = −0.65 and –0.57, *p* < 0.001) and best time (*r* = −0.70 and −0.60, *p* < 0.001) of the RSA. *Rf*_max_ was moderately associated with the relative peak power (*r* = 0.33, *p* = 0.011) of the WAnT and had small associations with total time (*r* = −0.26, *p* = 0.049) and best time (*r* = −0.27, *p* = 0.035) of the RSA. *S*_fv_ and *D*_rf_ were not associated with the WAnT or the RSA.

### Differences between male and female players

As shown in Table 2, independent Student *t*-tests demonstrated that male players were taller and heavier and performed better than female players in all variables except for *S*_fv_ and *D*_rf_. *F*_0_, *S*_fv_, and *Rf*_max_ of the male players were not associated with WAnT or RSA variables. *V*_0_ was moderately associated with peak power (*r* = 0.34, *p* = 0.037) and relative peak power (*r* = 0.34, *p* = 0.045) in the WAnT and was largely associated with total time (*r* = −0.59, *p* < 0.001) and best time (*r* = −0.64, *p* < 0.001) in the RSA. *P*_max_ was not associated with variables from the WAnT, but was moderately associated with total time (*r* = −0.41, *p* = 0.028) and largely associated with the best time (*r* = −0.53, *p* = 0.003) in the RSA. *D*_rf_ was moderately associated with total time (*r* = –0.39, *p* = 0.036) in the RSA.

**Table 2:** Male and female descriptive statistics and differences for *F*–*V*, WAnT, and RSA tests.

	Men	Women	*p*	ES ± 95%
*N*	36	34	n/a	n/a
Age	15.1 ± 0.2	16.5 ± 0.7	<0.001	−2.93 ± 0.68
Stature (cm)	177.5 ± 6.4	167.3 ± 5.5	<0.001	1.71 ± 0.55
Mass (kg)	70.6 ± 8.4	65.4 ± 7.0	0.007	0.67 ± 0.48
*F*_0_ (N kg^−1^)	7.30 ± 0.65	6.68 ± 0.80	<0.001	0.85 ± 0.49
*V*_0_ (m s^−1^)	8.43 ± 0.50	7.59 ± 0.40	<0.001	1.83 ± 0.57
*P*_max_ (W kg^−1^)	15.36 ± 1.67	12.69 ± 1.78	<0.001	1.55 ± 0.54
*S*_fv_ (N m s^−1^)	−0.87 ± 0.09	−0.88 ± 0.11	0.608	0.12 ± 0.47
*S*_fvopt_ (N m s^−1^)	−1.02 ± 0.04	−0.96 ± 0.04	<0.001	−1.58 ± 0.53
*FV*_imb._ (%)	14.92 ± 8.40	8.10 ± 8.92	0.002	0.79 ± 0.49
*RF*_max_ (%)	42.38 ± 3.01	39.27 ± 4.26	<0.001	0.85 ± 0.5
*D*_rf_ (%)	−8.15 ± 0.86	−8.38 ± 0.96	0.279	0.26 ± 0.47
Peak power (W)	883.69 ± 119.95	712.50 ± 90.87	<0.001	1.60 ± 0.54
Relative peak power (W kg^−1^)	8.79 ± 0.47	6.90 ± 0.70	<0.001	3.17 ± 0.72
Average power (W)	621.19 ± 76.45	447.62 ± 61.67	<0.001	2.49 ± 0.65
Relative average power (W kg^−1^)	6.20 ± 0.47	4.36 ± 0.64	<0.001	3.30 ± 0.73
Anaerobic capacity (W)	3,672.61 ± 455.34	2,676.29 ± 339.41	<0.001	2.47 ± 0.63
Anaerobic fatigue (%)	47.69 ± 6.64	52.38 ± 7.35	0.007	0.67 ± 0.48
Total time (s)	45.42 ± 1.40	48.73 ± 1.70	<0.001	−2.01 ± 0.61
Best time (s)	7.31 ± 0.24	7.77 ± 0.28	<0.001	−1.65 ± 0.58
Percentage decrement score (%)	3.53 ± 1.92	4.50 ± 1.84	0.010	−0.68 ± 0.52

ES, Effect size.

**Figure 1:**
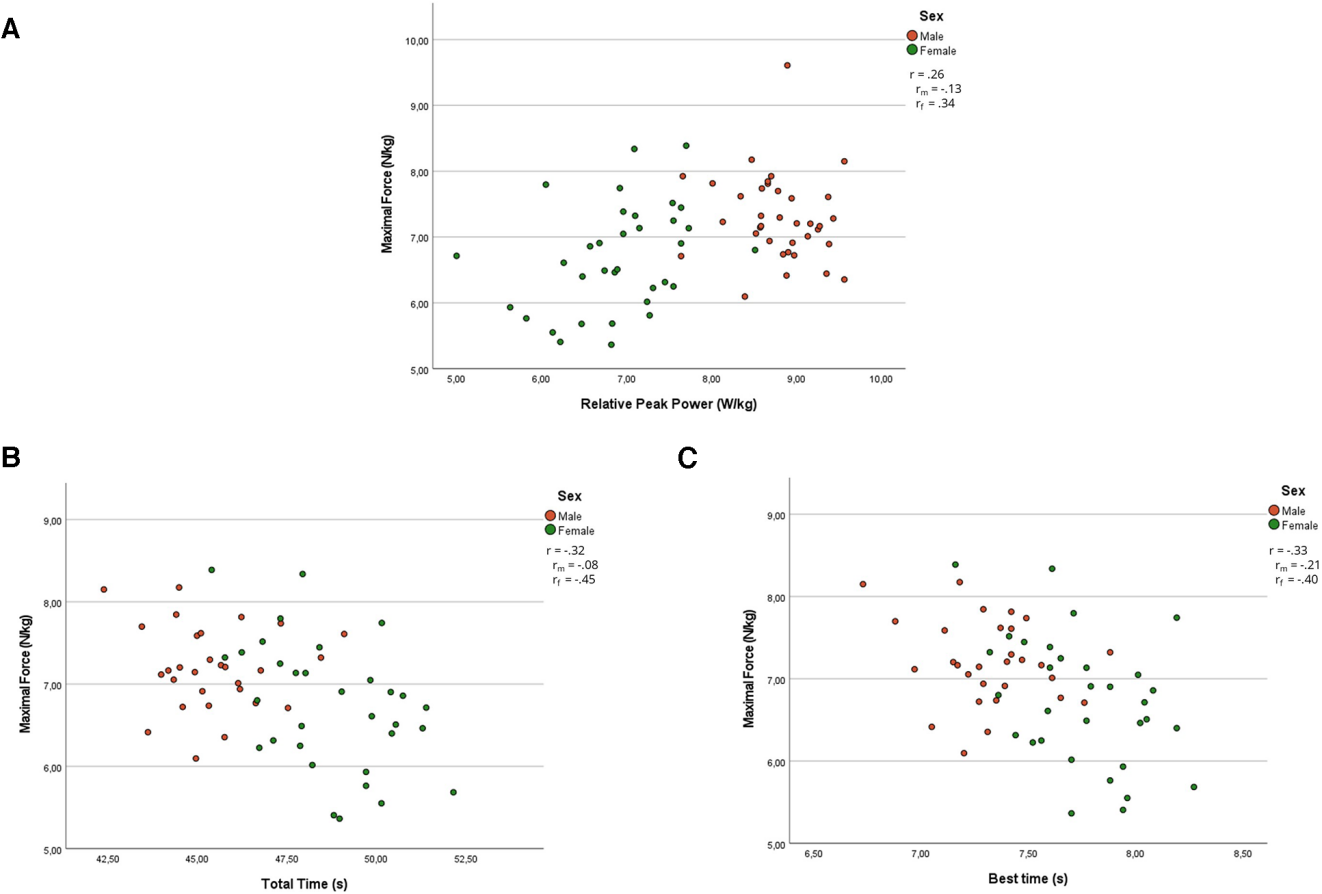
Partial correlation and correlations between maximal theoretical force (*F*_0_) and relative peak power (**A**), total time (**B**), and best time (**C**) for pooled data and male and female data, respectively. *r*, partial correlation; *r*_m_, correlation for men; *r*_f_, correlations for women.

**Figure 2:**
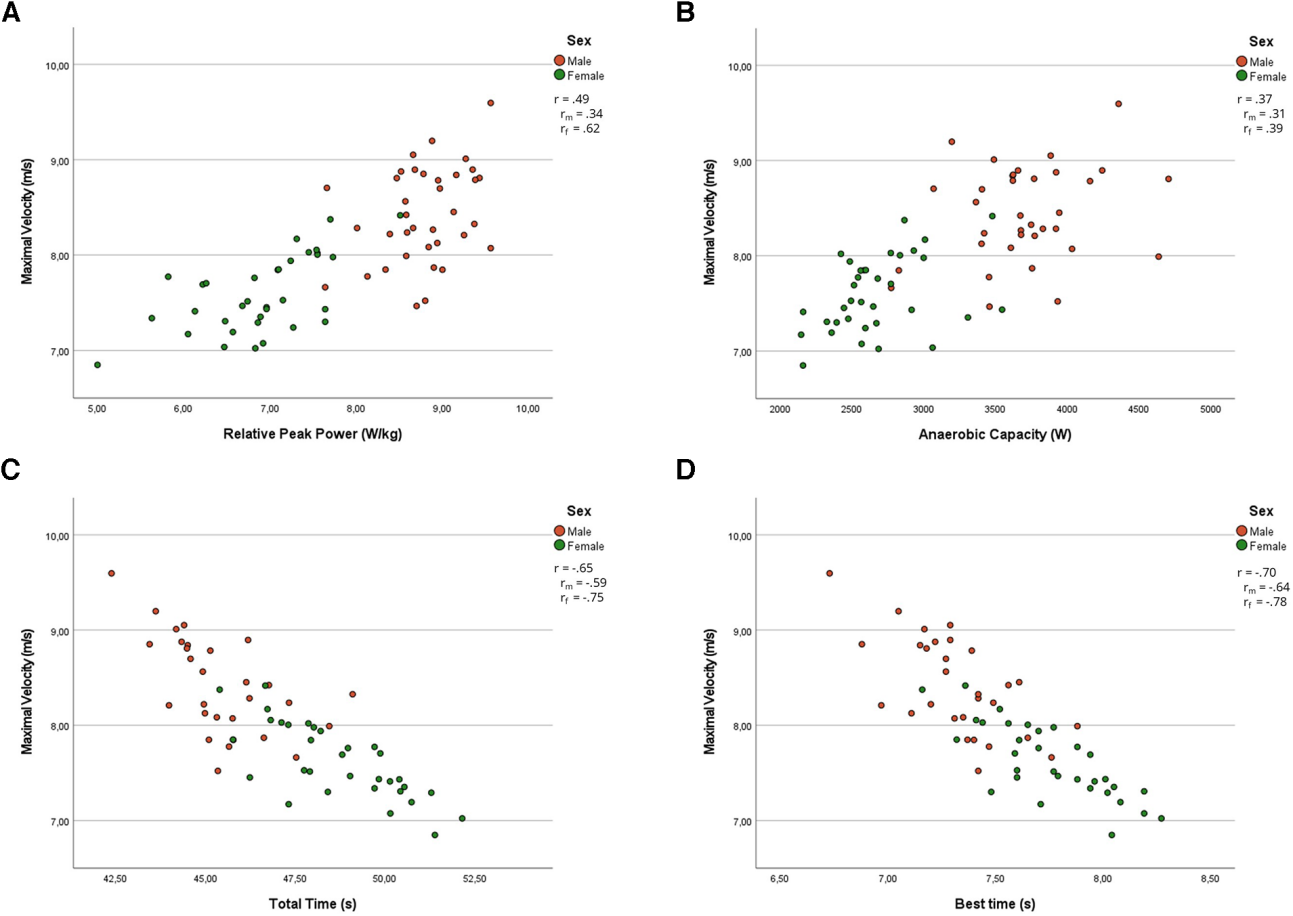
Partial correlation and correlations between maximal theoretical velocity (*V*_0_) and relative peak power (**A**), anaerobic capacity (**B**), total time (**C**), and best time (**D**) for pooled data and male and female data, respectively. *r*, partial correlation; *r*_m_, correlation for men; *r*_f_, correlations for women.

*S*_fv_ and *D*_rf_ of the female players were not associated with WAnT or RSA variables. *F*_0_ was moderately associated with relative peak power (*r* = 0.34, *p* = 0.047), relative average power (*r* = 0.34, *p* = 0.050) of the WAnT, and total time (*r* = −0.45, *p* = 0.010) and best time (*r* = −0.40, *p* = 0.022) of the RSA. *V*_0_ was largely associated to relative peak power (*r* = 0.62, *p* < 0.001), moderately associated with peak power (*r* = 0.47, *p* = 0.005) and anaerobic capacity (*r* = 0.39, *p* = 0.022) of the WAnT, and largely associated with total time (*r* = −0.75, *p* < 0.001) and best time (*r* = −0.78, *p* < 0.001) of the RSA. *P*_max_ was largely associated with relative peak power (*r* = 0.53, *p* = 0.001), moderately with relative average power (*r* = 0.38, *p* = 0.026) of the WAnT, and largely associated with total time (*r* = −0.68, *p* < 0.001) and best time (*r* = −0.66, *p* < 0.001) of the RSA. *Rf*_max_ was moderately associated with relative peak power (*r* = 0.48, *p* = 0.004), relative average power (*r* = 0.38, *p* = 0.025) of the WAnT and with total time (*r* = −0.43, *p* = 0.013), and best time (*r* = −0.45, *p* = 0.011) of the RSA. Partial correlations and correlations for male and female players for *F*_0_ and *V*_0_ are illustrated in Figures 1, 2 to express the main differences between the sexes.

## Discussion

The relevance of *F*–*V* athlete profiling has emerged over the last decade. However, little is known about its associations with physical attributes that require high-intensity efforts, such as anaerobic capacities. Considering the importance of such qualities to excel in ice hockey, a deeper understanding of these associations is helpful for strength and conditioning coaches to optimize performance. This paper was the first to explore the associations between sprinting *F*–*V* mechanical capabilities and anaerobic capacities of highly competitive adolescent male and female ice hockey players. The general aim of this study was to widen current knowledge around sprinting *F*–*V* and its links with anaerobic capacities measured threw common tests. Previous work analyzing the sprinting *F*–*V* of ice hockey players was fairly limited focusing more on the validity and reliability of the method with a limited number of subpopulations, that is, female French senior to junior national teams and male players from elite Finnish leagues (16–19). The first objective of this study was to explore how the mechanical capabilities of sprinting *F*–*V* were related to variables from common anaerobic tests such as the Wingate and repeated-sprint test. To provide a complete observation of these associations, three main hypotheses were proposed. The first hypothesis was that *P*_max_ would show the strongest associations with the different variables of both anaerobic tests, *V*_0_ would show stronger associations with the cycling test, and *F*_0_ would show stronger associations with the repeated-sprint ability. When considering results for pooled data, *P*_max_ was associated with anaerobic power and anaerobic capacity in both tests yet appeared stronger for the running test (moderate associations for peak power, relative peak power, and average power in the WAnT vs. large associations for total time and best time in the RSA). Unlike initially hypothesized, *V*_0_ showed the strongest associations with performance in both tests, correlating largely with the RSA and moderately with the WAnT. In contrast, *F*_0_ showed few associations to the Wingate with a small association to relative peak power and moderate associations with the RSA. The initial hypothesis was based on the proposed interpretations of Morin and Samozino (15) and only focused on an analysis of the tasks used in this study. However, these interpretations were majorly based on the observations with elite-level athletes. The different playing levels and ages of participants from this study probably influenced the associations observed in this study.

**Figure 3:**
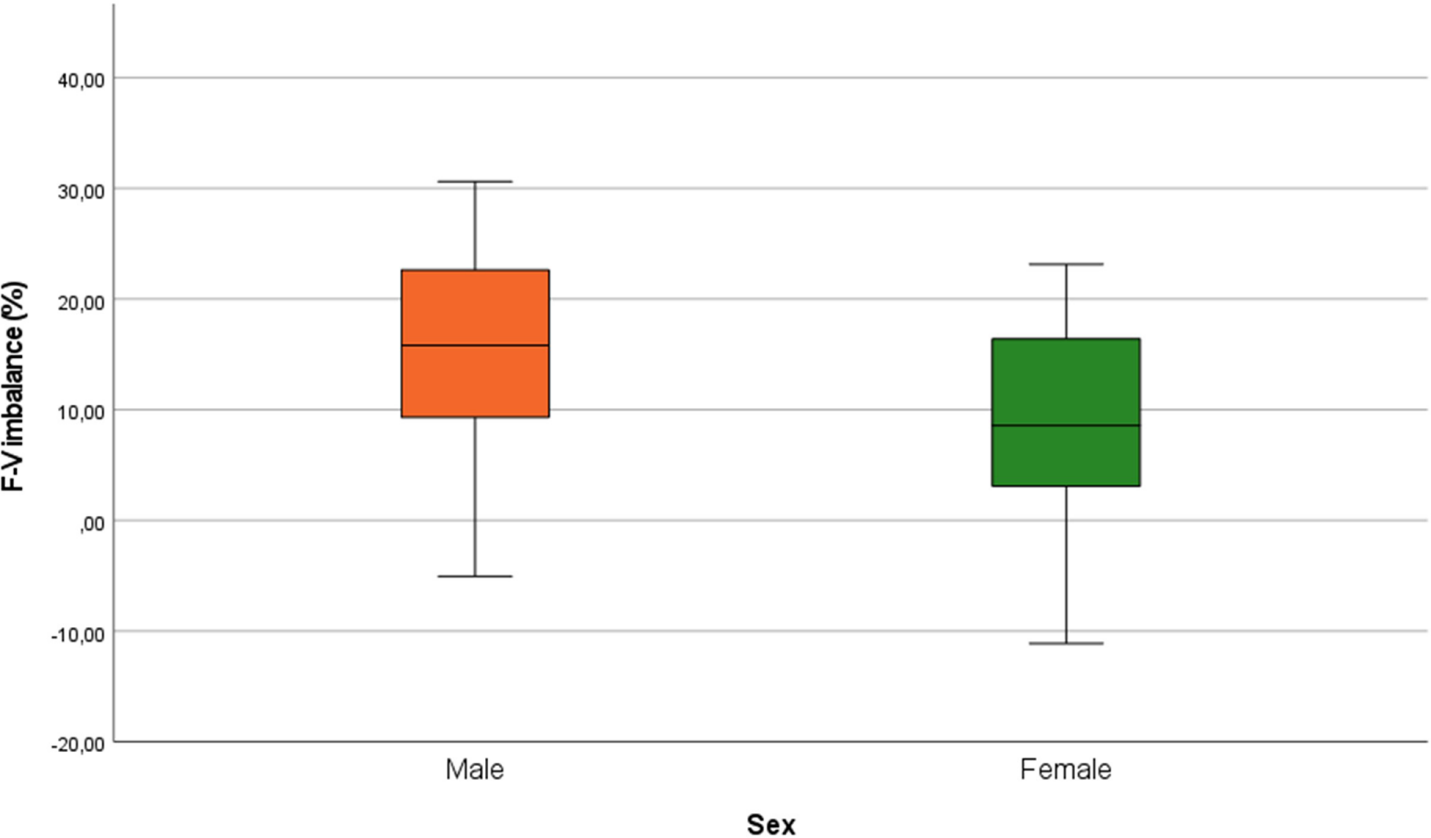
Box plot of *F*–*V* imbalances for male and female players.

The second objective of this study was to explore differences between male and female players. The hypothesis was that *F*_0_ would exhibit stronger associations with performance in female athletes as they tend to have more force-oriented *F*–*V* profiles (27, 28). Our results confirmed this hypothesis as associations between *F*_0_ and performance in both anaerobic tests were only present for the female players (see Figures 1, 2). The cycling performance and repeated-sprint performance of male players in this study were mainly determined by *V*_0_. Given their young age, *F*_0_ may not have been fully developed at the moment of testing (22) and could explain the observed differences with female players. Previous work by Perez et al. (16) highlighted the absence of associations between the actual *F*–*V* and performance of an athlete as observed in this study. To our knowledge, this study was the first to include the computation of *S*_fvopt_ and *FV*_imb._ in the evaluation of the sprinting *F*–*V* with ice hockey players. Looking at Figure 3, both groups show a force deficit which may explain the large associations between *V*_0_ and RSA performance. The appearance of associations with *F*_0_ for female players could be attributed to the portion of players with a velocity deficit (bottom quartile of female box plot). Nonetheless, practitioners training sprint ability and anaerobic capacities concurrently should choose training methods according to the sex of players. Highly trained female players may benefit from methods including explosive exercises at low and high velocities while highly trained male players in the age range of this study could focus more on developing maximal velocity. Jiménez-Reyes et al. (12) categorized exercises according to the desired training focus for enhanced jumping performance. Similarly, practitioners could use resisted sprints for an emphasis on low velocities (5) and flying sprints to train at higher velocities to enhance sprint performance.

Although this study adds to available knowledge regarding sprinting *F–V* profiling in ice hockey, certain limitations persist. First, while the reliability of the computation method by Samozino et al. (14) has been reported with youth athletes (22), no validation of the method has been published. The sprinting mechanics of teenage ice hockey players are different from elite adult sprinters. However, participants in this study have exhibited lower mechanical characteristics than higher-level athletes in previous studies (17–19). Given velocity follows the same mono-exponential function in youth (22) and aerodynamics included in the method adjusts to the stature and mass of each individual (14), there are a few reasons that the method should not be valid with this study population. Second, the observational design of this study does not confirm the causal effects of the different sprinting *F*–*V* variables on anaerobic performance. The type and strength of associations could be explained by the theoretical basis presented by Morin and Samozino (15) and previous observations. However, further research should focus on interventions demonstrating the advantages of individualized training based on the *F*–*V* as this would yield the best practical applications (12, 39). Third, this study only considered off-ice data that were collected during off-season. Prediction of skating abilities from off-ice activities or the transfer of such activities to skating performance and anaerobic capacities on the ice remains unclear. For example, Perez et al. (16) demonstrated that sprinting and skating *F*–*V* were not correlated. Ultimately, the goal of ice hockey training is to maximize performance on the ice. In this regard, developing research designs that allow the evaluation of the transferability of off-ice attributes to on-ice performance is a key concern for stakeholders overseeing the development of highly competitive ice hockey players. Comparing the skating *F*–*V* and anaerobic capacities of players on the ice would allow for a better understanding of determinant indicators in ice hockey and designing efficient training programs.

## Conclusion

This research provides a deeper understanding of underlying associations between the sprinting *F*–*V* and anaerobic capacities of highly trained adolescent male and female ice hockey players. Pooled data demonstrated that the ability to apply force at low and high velocities are determinant for anaerobic performance on a cycle ergometer and repeated-sprint ability including changes of direction. A separate analysis of male and female players demonstrated that determinant mechanical capabilities of sprinting vary according to sex. Specifically, the cycling and repeated-sprint performance of younger male players could be mainly determined by the ability to apply force at high velocities, whereas the ability to generate force at slow speeds may be of added importance for female players. Practitioners must understand that determinant mechanical capabilities of sprinting for anaerobic performance may be different for male and female athletes and training priorities may change accordingly.

## Data Availability

The raw data supporting the conclusions of this article will be made available by the authors, without undue reservation.
